# Metabolic Biomarkers of Monochorionic Twins Complicated With Selective Intrauterine Growth Restriction in Cord Plasma and Placental Tissue

**DOI:** 10.1038/s41598-018-33788-y

**Published:** 2018-10-29

**Authors:** Lianlian Wang, Ting-Li Han, Xiaofang Luo, Siming Li, Tim Young, Chang Chen, Li Wen, Ping Xu, Yangxi Zheng, Richard Saffery, Philip N. Baker, Chao Tong, Hongbo Qi

**Affiliations:** 1 Department of Obstetrics, the First Affiliated Hospital of Chongqing Medical University, Chongqing, 400016 China; 2 International Collaborative Joint Laboratory of Reproduction and Development of Ministry of Education P.R.C, Chongqing Medical University, Chongqing, 400016 China; 3 State Key Laboratory of Maternal and Fetal Medicine of Chongqing Municipality, The First Affiliated Hospital of Chongqing Medical University, Chongqing, 400016 China; 4 Department of Reproduction Health and Infertility, the First Affiliated Hospital of Chongqing Medical University, Chongqing, 400016 China; 5 Liggins Institution, University of Auckland, Auckland, 1142 New Zealand; 6 Faculty of Health and Environmental Sciences, Auckland University of Technology, Auckland, 1010 New Zealand; 7 Institute of Life Sciences, Chongqing Medical University, Chongqing, 400016 China; 8 Department of Paediatrics, University of Melbourne, Parkville, VIC Australia; 9 College of Life Sciences, University of Leicester, Leicester, LE1 7RH UK

## Abstract

The selective intrauterine growth restriction (sIUGR) of monochorionic diamniotic (MCDC) twins causes phenotypic growth discordance, which is correlated with metabolomic pertubations. A global, untargeted identification of the metabolic fingerprint may help elucidate the etiology of sIUGR. Umbilical cord blood and placentas collected from 15 pairs of sIUGR monochorionic twins, 24 pairs of uncomplicated twins, and 14 singletons diagnosed with intrauterine growth restriction (IUGR) were subjected to gas chromatography-mass spectrometry based metabolomic analyses. Supervised multivariate regression analysis and pathway analysis were performed to compare control twins with sIUGR twins. A generalized estimating equation (GEE) model was utilized to explore metabolic differences within sIUGR co-twins. Linear logistic regression was applied to screen metabolites that significantly differed in concentration between control twins and sIUGR twins or IUGR singletons. Umbilical cord blood demonstrated better global metabolomic separation of sIUGR and control twins compared to the placenta. Disrupted amino acid and fatty acid metabolism as well as high levels of exposure to environmental xenobiotics were associated with sIUGR. The metabolic abnormalities in MCDA twins suggested that *in utero* growth discordance is caused by intrauterine and extrauterine environmental factors, rather than genetics. Thus, this study provides new therapeutic targets and strategies for sIUGR management and prevention.

## Introduction

The World Health Organization has stated that the prevention of low birth weight and reduction of infant mortality have been two of the most important long-term public health priorities from previous decades until now^1^. However, our current understanding of the underlying metabolic mechanisms leading to suboptimal growth remains limited. Selective intrauterine growth restriction (sIUGR) is one of the most serious complications of monochorionic diamniotic (MCDA) twins with an incidence of 10–25%^2–4^. Compared with singleton intrauterine growth restriction (IUGR), sIUGR presents higher risks of adverse pregnancy and postpartum outcomes for both the mother and offspring^5,6^. Most importantly, compromised intrauterine growth has been associated with long-term health issues in adulthood^7–9^.

Although great endeavors have elucidated that fetal growth restriction may be driven by both genetic and environmental factors^10,11^, the underlying molecular mechanism remains largely unknown. However, clinicians have found that morphological abnormality does not apply to all sIUGR cases^12,13^. Meanwhile, emerging evidence suggests that sIUGR is associated with impaired placental transportation of amino acids^14^. Maternal nutrient deficiencies can also cause a suboptimal intrauterine environment and metabolic shifts, thus shifting the fetal metabolome (collection of detectable low-weight biochemical intermediates known as metabolites), which may further influence fetal gene expression via epigenetic modification^6^.

Metabolomics is the analysis of metabolites and has evolved into a powerful diagnostic and predictive tool in many scientific disciplines^15^, especially for the study of phenotypes with complex etiologies^16^. Metabolomics enables the investigation of overall metabolic alterations as a disease is initiated and progresses, thus allowing for the identification of prognostic biomarkers at early stages of disease development. Despite the merits of metabolomic analysis, studies utilizing metabolomic technologies to unveil the pathophysiology of sIUGR are rare^17^.

sIUGR twins are the best model to investigate the pathophysiology underpinning fetal growth impairment of humans, as monochorionic twins have identical genotypes; therefore, the discordance in their intrauterine growth can be attributed to differences in non-shared environments^18^. In addition, comparing sIUGR twins with IUGR singletons may reveal common mechanisms of suboptimal intrauterine growth.

In this study, we performed comprehensive metabolomic profiling of the umbilical cord blood and placental tissue from MCDA sIUGR twins and normally grown MCDA twins as well as IUGR singletons. Our results demonstrated that phenotypic discordance in sIUGR twins was related to altered essential amino acid composition, disrupted amino acid biochemical pathways and fatty acid metabolism.

## Results

### Population characteristics

Maternal characteristics and pregnancy outcomes of the sIUGR and control MCDA pregnancies are described in Table 1. The maternal age, occupation, body mass index (BMI), primigravida, smoking status, mode of conception, gestational age at delivery, delivery method, and neonatal sex are included. The histopathological assessments of placenta were summarized in Tables S1 and S2. Table 2 details the postnatal outcomes in sIUGR and control MCDA twins. In these two groups, the amniotic fluid volume and Apgar score (1, 5, and 10 min) were similar between co-twins in both groups, while the birth weight was significantly different within sIUGR co-twins (p < 0.002). The placenta weight and placenta volume were similar between the sIUGR and control MCDA groups, while the birth weight discordance within co-twins was significant (p < 0.001). In addition, the maternal age, occupation status, BMI, gravidity, smoking status, delivery method, and neonatal sex were not significantly different between IUGR and sIUGR pregnancies (Table 3). However, the average gestational age at delivery was 38 weeks in the IUGR group and 35 weeks in the sIUGR group (p < 0.001). The sIUGR group also had a higher placenta weight (p < 0.001) and placenta volume (p < 0.01) than the IUGR group. Nevertheless, the average birth weight of the smaller sIUGR co-twin was significantly lower than that in the IUGR group (p = 0.02).

**Table 1:** Comparison of clinical characteristics in the control twin and sIUGR twin groups.

Characteristics and pregnancy outcomes	sIUGR group (n = 15)	Control group (n = 24)	P-Value
Maternal age (years)	30.0 ± 3.5	29.0 ± 3.7	0.20^a^
**Employed outside the home**			0.91^c^
Yes	9 (60%)	13 (54.2%)	
No	6 (40%)	11 (45.8%)	
Body mass index (kg/m^2^**)**	21.0 ± 2.5	21.0 ± 2.8	0.94^a^
Primigravida	9/15 (60%)	13/24 (54.2%)	0.98^c^
Smoking during pregnancy	0/15 (0%)	0/24 (0%)	1.00
**IVF-ET/natural conception**			1.00^c^
IVF-ET^e^	1 (6.7%)	2 (8.3%)	
Natural conception	14 (93.3%)	22 (91.7%)	
Gestational age at delivery (wks)	35 (34, 37)	36.5 (35, 37)	0.07^d^
**Delivery**			1.00^d^
Cesarean	15 (100%)	24 (100%)	
Vaginal	0 (0%)	0 (0%)	
**Neonatal sex**			0.78^c^
Male/male	9 (60%)	12 (50%)	
Female/female	6 (40%)	12 (50%)	

^a^Student’s T-test. ^b^Mann-Whitney U test. ^c^Chi-square test. ^d^Fisher’s exact test; *p < 0.001. ^e^IVF-ET, *In vitro* fertilization & embryo transfer.

**Table 2:** Comparison of postnatal outcomes in the control twin and sIUGR twin groups.

Postnatal outcomes	sIUGR group			Control group		
	T1 (n = 15)	T2 (n = 15)	P-Value	T1 (n = 24)	T2 (n = 24)	P-Value
Birth weight (g)	2560 ± 485	1920 ± 454	0.002*^a^	2675 ± 311	2518 ± 295	0.08^a^
Amniotic fluid volume (ml)	500 (500, 600)	500 (400, 560)	0.41^b^	600 (500, 1000)	500 (500, 600)	0.13^b^
Apgar score at 1 min	9 (9, 10)	9 (9, 10)	0.91^b^	10 (9, 10)	10 (9, 10)	0.77^b^
Apgar score at 5 min	10 (10, 10)	10 (10, 10)	0.53^b^	10 (10, 10)	10 (10, 10)	1^b^
Apgar score at 10 min	10 (10, 10)	10 (10, 10)	0.31^b^	10 (10, 10)	10 (10, 10)	1^b^
Birth weight discordance (g)	530 (470, 560)			160 (90, 225)		9.22 × 10^−7^*^b^

^a^Student’s T-test. ^b^Mann-Whitney U test; *P < 0.05. T1, twin 1; T2, twin 2.

**Table 3:** Comparison of the clinical characteristics of sIUGR twins and IUGR singleton pregnancies.

Characteristics	sIUGR group (n = 15)	IUGR group (n = 14)	P-Value
Maternal age (years)	30 ± 4	30 ± 4	0.62^a^
Body mass index (kg/m^2^)	21.0 ± 2.5	21.7 ± 2.7	0.53^a^
Primigravida	9/15 (60%)	8/15 (53%)	0.58^c^
Smoking	0/15 (0%)	0/14 (0%)	1.00^d^
Gestational age at delivery (wks)	35 (34, 37)	38 (37, 38)	0.0004^b^**
**Delivery**			1.00^b^
Cesarean	15 (100%)	14 (93.3%)	
Vaginal	0 (0%)	1 (6.7%)	
**Neonatal sex**			0.14^c^
Male	9 (60%)	4 (26.7%)	
Female	6 (40%)	11 (73.3%)	
Placenta weight (g)	656.92 ± 96.12	470.67 ± 81.80	1.29 × 10^−5^ ^a^**
Placenta volume (cm^3^)	962.34 ± 263.63	631.32 ± 226.61	0.002^a*^
Average birth weight (g)	(2560 ± 486, 1920 ± 454)^e^	2253 ± 264	(0.05, 0.02^*^)^a,f^

^a^Student’s T-test. ^b^Mann-Whitney U test. ^c^Chi-square test. ^d^Fisher’s exact test; *P < 0.001; *P < 0.05. ^e^The first value is the average birth weight of the sIUGR larger twin, and the second value is the average birth weight of the sIUGR smaller twin. ^f^The first P-value is the significance of the birth weight difference between the IUGR singleton and the sIUGR larger twin; the second value is the significance of the birth weight difference between IUGR singleton and the sIUGR smaller twin.

### Metabolome of umbilical cord plasma and placental tissue of control twin pairs and sIUGR twin pairs

Over 200 individual spectral peaks were separated by gas chromatography, from which 79 umbilical cord blood plasma metabolites and 84 placental metabolites (Table S3) were confidently curated using our in-house MCF and commercial National Institute of Standards and Technology (NIST) spectral libraries^19^.

### PLSDA analysis of metabolites in umbilical cord plasma from control and sIUGR twins

**Figure 1:**
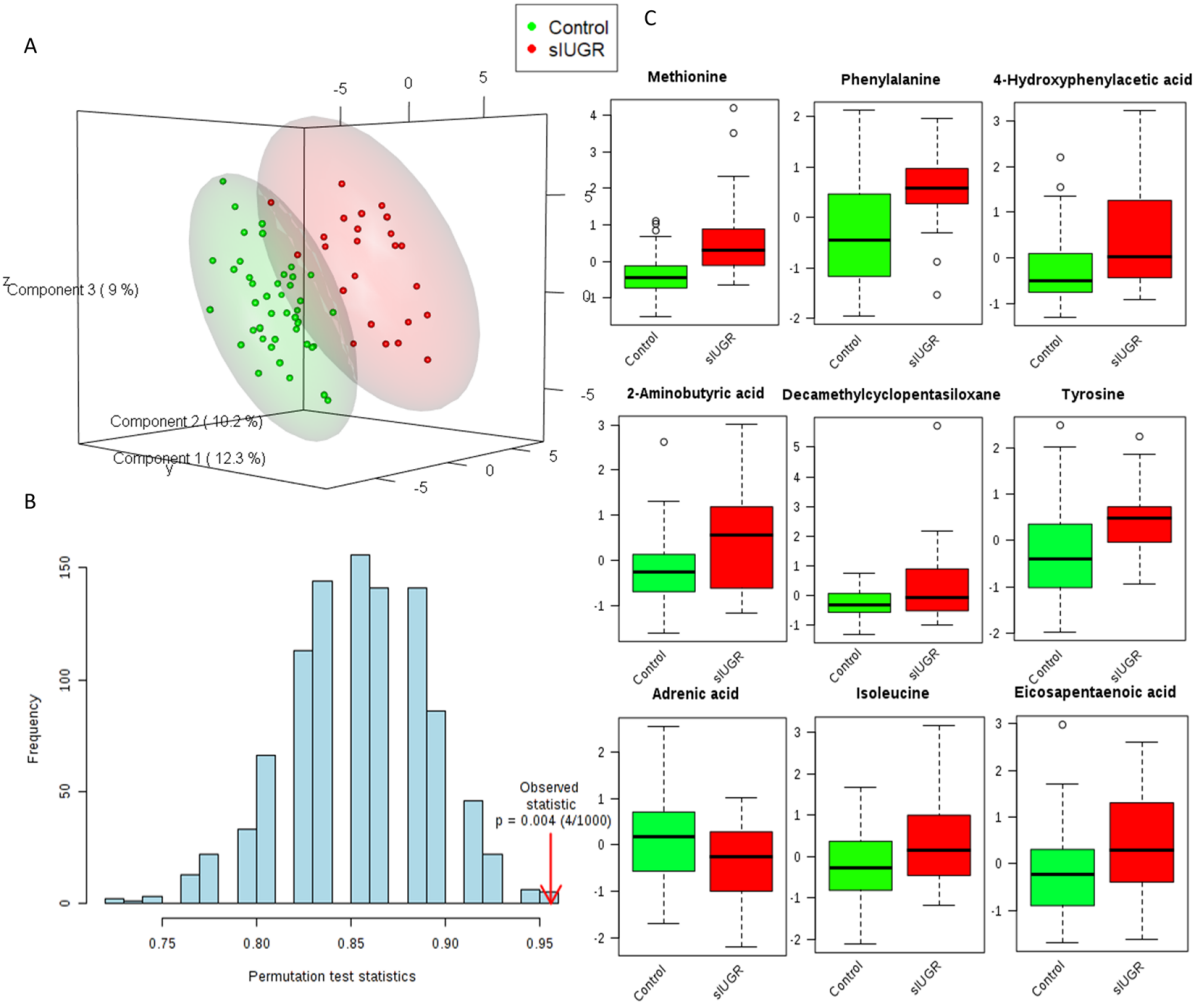
Supervised multivariate classification analysis of the umbilical cord plasma metabolite profiles of control twins and sIUGR twins. (**A**) Score plot of the partial least squares discriminant analysis (PLS-DA). (**B**) Validation of the PLS-DA model using permutation. The results are shown as p-values. The blue bars represent the accuracy of the prediction frequencies. (**C**) Boxplots showing the nine most significant metabolites based on their variable importance in projection scores from the PLS-DA model with their relative concentrations. The green boxes represent the control group, while the red boxes represent the sIGUR group. Unknown 355(100) 73(67.0) 267(68.7) (NIST: Cyclopentasiloxane, decamethyl-, Match: 928, R. Match: 928, Prob: 94.4%).

PLS-DA demonstrated that metabolites in the umbilical cord plasma samples from control twins and sIUGR twins were clustered separately (Fig. 1A). The first three latent variables accounted for 12.3%, 10.2%, and 9% of the variation in metabolite levels between control and sIUGR twins. The performance of the PLS-DA model was evaluated using prediction accuracy during model training, which was permutated 1000 times (Fig. 1B). The results showed a confidence level of model accuracy (p = 0.004). In addition, the PLS-DA model inferred which metabolites were most important for classification according to their VIP scores (VIP scores > 1.0) (Fig. 1C). The top 9 significant metabolites that contributed to the separation of the control twins and sIUGR twins were ranked, and their relative concentrations in control and sIUGR twins are provided in Fig. 1C. Among these metabolites, essential amino acids, such as methionine, phenylalanine and tyrosine, were ranked highly according to their VIP scores. In addition, a few unannotated metabolites (labeled as unknown) were also notable.

### PLS-DA for metabolites in placental tissue from control and sIUGR twins

**Figure 2:**
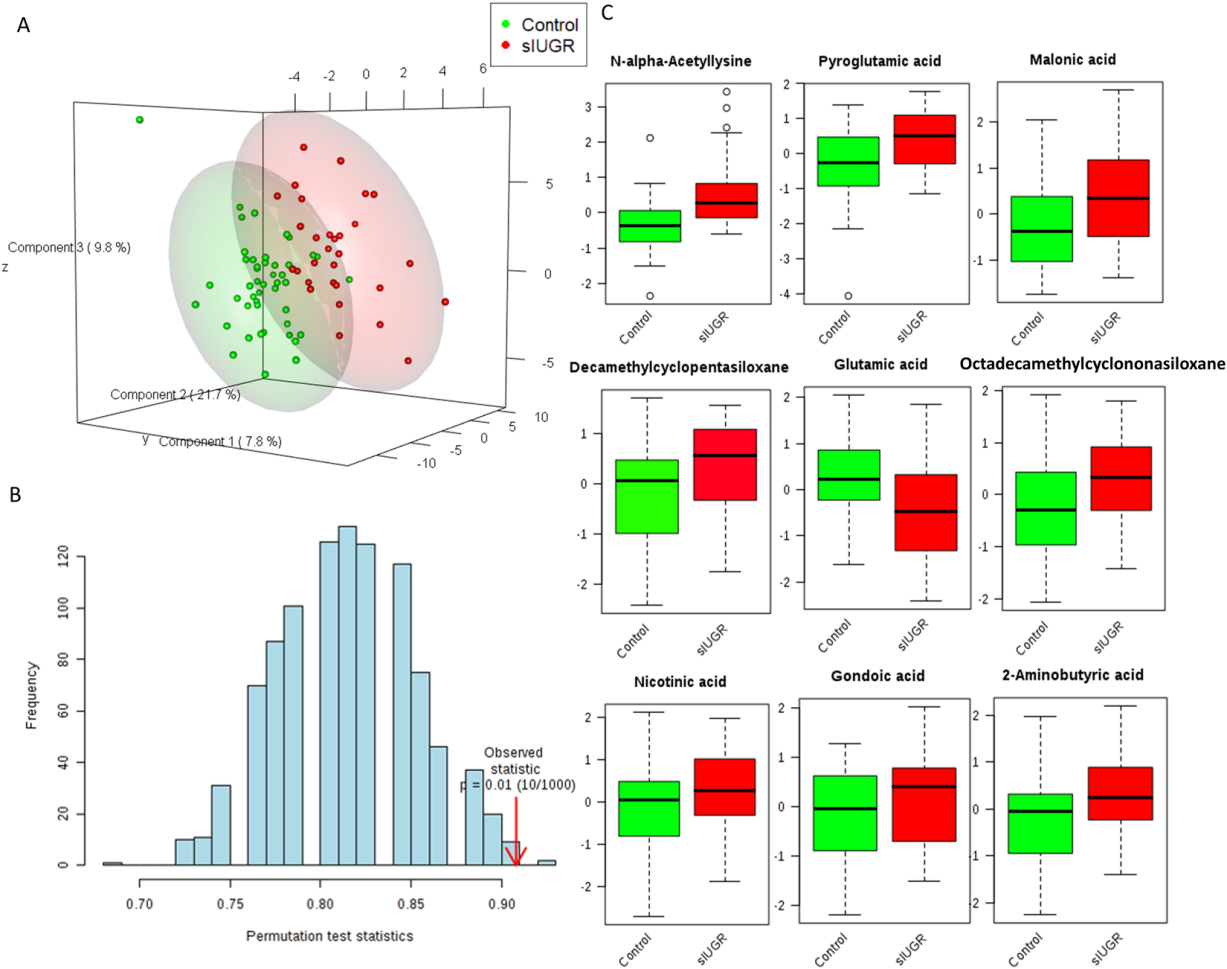
Supervised multivariate classification analysis of the placenta metabolite profiles of control twins and sIUGR twins. (**A**) Score plot of the partial least squares discriminant analysis (PLS-DA). (**B)** Validation of the PLS-DA model using permutation. The results are shown as p-values. The blue bars represent the accuracy of the prediction frequencies. (**C)** Boxplots showing the nine most significant metabolites based on their variable importance in projection scores from the PLS-DA model with their relative concentrations. The green boxes represent the control group, while the red boxes represent the sIGUR group. Unknown 074(100) 429(77.4) 355(44.6) (NIST: Cyclononasiloxane, octadecamethyl, Match: 908, R. Match:910, Prob: 95.2%). Unknown 355(100) 73(67.0) 267(68.7) (NIST: Cyclopentasiloxane, decamethyl, Match: 935, R. Match: 935, Prob: 94.3%).

Metabolites identified in placental samples from control twins and sIUGR twins were also separately clustered as shown by the PLS-DA score plot (Fig. 2A). The first three latent variables accounted for 21.7%, 9.8%, and 7.8% of the variation in metabolite levels between control and sIUGR twins. The performance of the model was evaluated using prediction accuracy during model training, which was permutated 1000 times (Fig. 2B). The results showed a confidence level of model accuracy (p = 0.01). Our data suggested that compared to the placenta, the umbilical cord plasma PLS-DA model showed better separation of the metabolic profile between the control twins and sIUGR twins. The top 9 significant metabolites that contributed to the separation of the placenta model between the control twins and sIUGR twins were ranked (Fig. 2C) (VIP scores > 1.0). Among these metabolites, the lysine derivative n-alpha-acetyl lysine, pyroglutamic acid, and glutamic acid had high VIP scores, as did six unannotated metabolites.

### Pathway analysis and quantitative enrichment analysis of control twins and sIUGR twins

**Figure 3:**
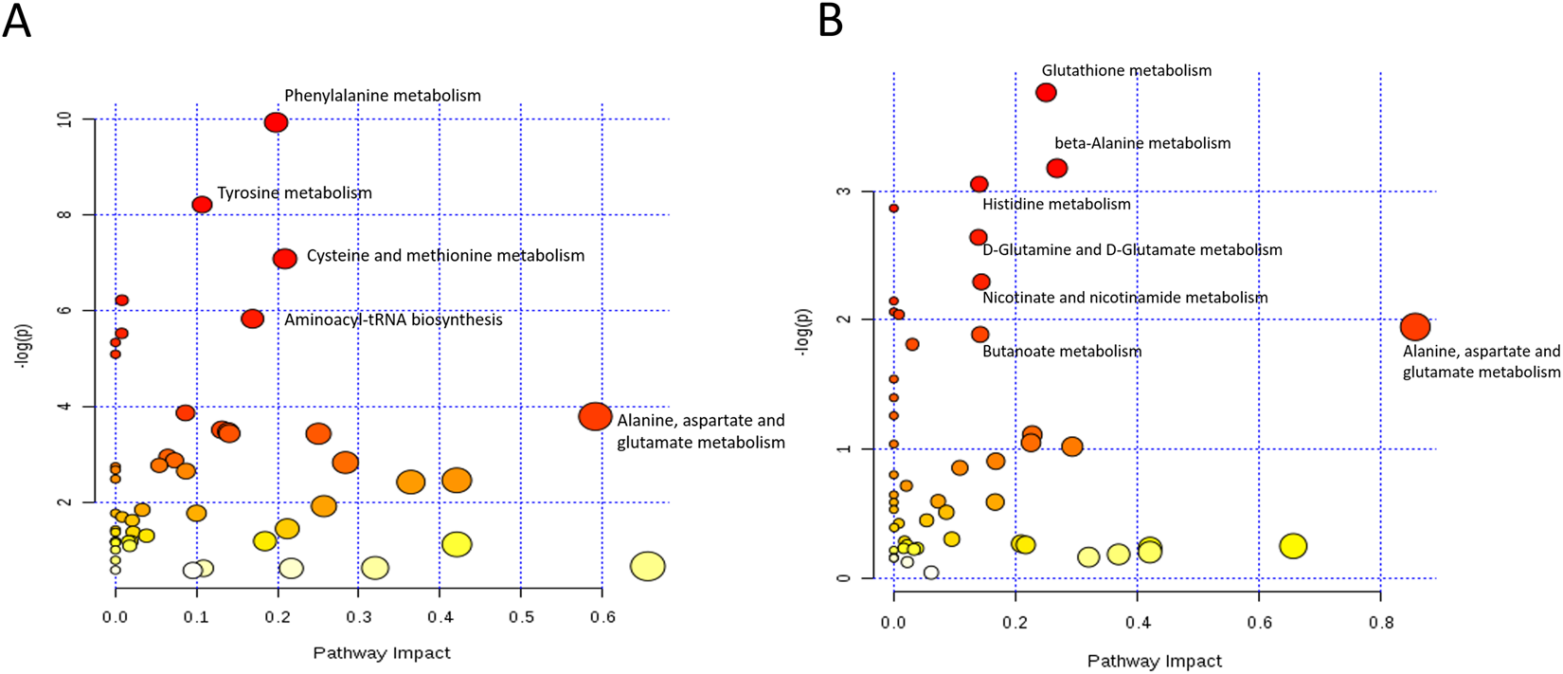
Topological pathway analysis of the umbilical cord plasma (left) and placenta (right) metabolites of control twins and sIUGR twins. The most significant metabolic pathways are indicated by the color and size of the spheres (red = most significant, yellow = least significant) according to their p-values and statistical pathway impact values, analyzed by quantitative enrichment analysis (QEA).

Metabolic pathway analysis based on the collection of annotated metabolites from the umbilical cord plasma and placenta samples revealed the most pertinent pathways that appear to be associated with sIUGR (Fig. 3). Forty-three pathways containing at least 2 significantly differentially annotated metabolites between the two twin groups were recognized by the KEGG database. For umbilical cord plasma samples, pathways containing more than two of the detected metabolites with QEA p-values and quantitative enrichment analysis (QEA) false discovery rate (FDR) values less than 0.05 and a pathway impact value of greater than 0.1 were categorized as potential pathways of interest. According to the abovementioned selection criteria, eight pathways were identified as disturbed in sIUGR twins compared to those in control MCDA twins; these pathways included phenylalanine metabolism, tyrosine metabolism, cysteine and methionine metabolism, tryptophan biosynthesis, aminoacyl-tRNA biosynthesis, nitrogen metabolism, ubiquinone and other terpenoid-quinone biosyntheses, and thiamine metabolism (Table S4). On the other hand, none of the 43 pathways identified in the placental samples met the criterion of having a QEA FDR value lower than 0.05. Nevertheless, three metabolic pathways — glutathione metabolism, beta-alanine metabolism, and histidine metabolism — had a QEA p-value less than 0.05 (Table S5), indicating potential disruption in the sIUGR placenta.

### Correlation of metabolites with weight discordance within sIUGR co-twins

**Figure 4:**
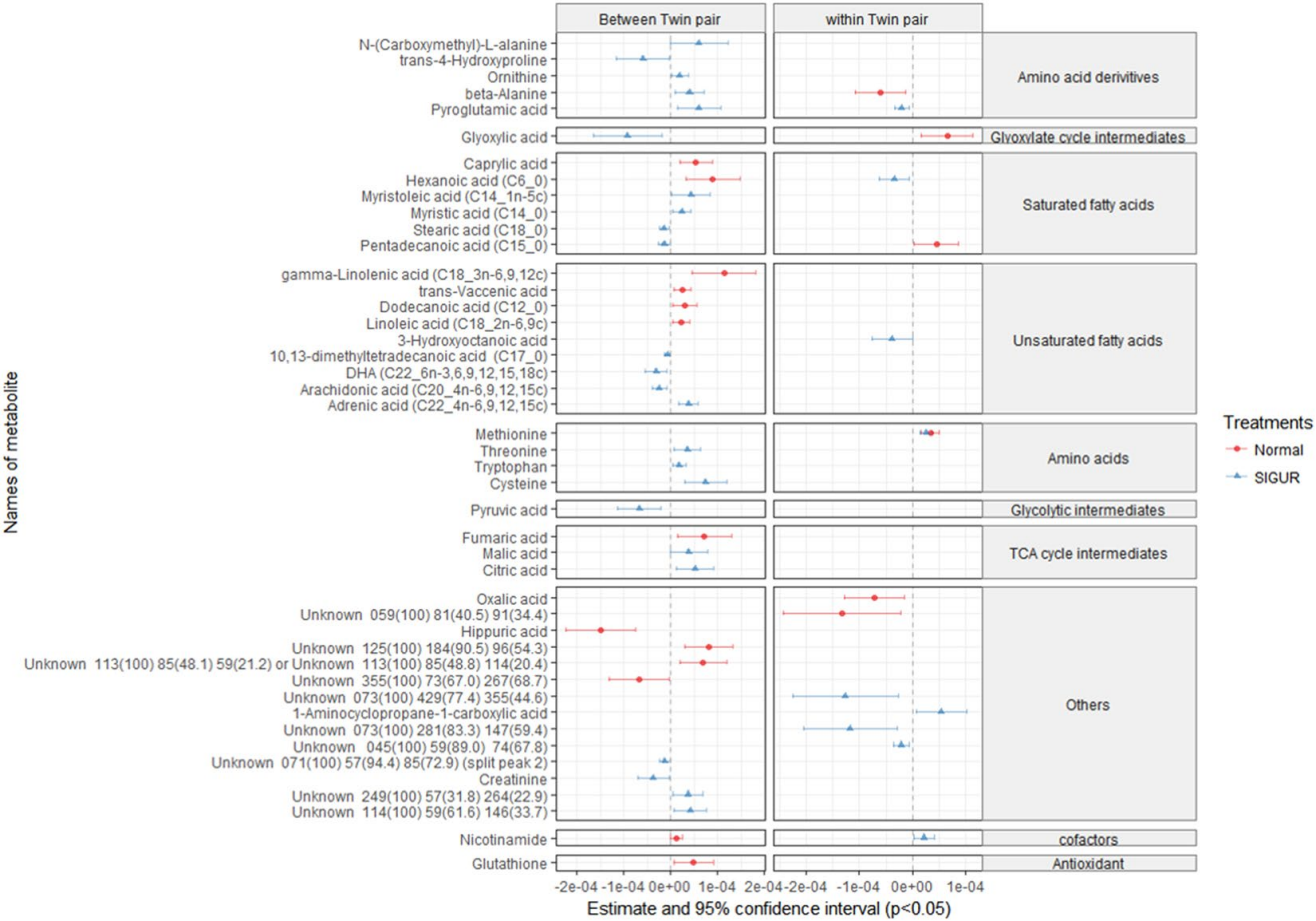
Correlation of birth weight discordance within and between twin pairs of umbilical cord plasma metabolites detected from sIUGR and normal twins, analyzed using a generalized estimating equation (GEE). The red lines represent the 95% confidence intervals for the correlation of metabolites with weight discordance in the control umbilical cord plasma. The blue lines represent the 95% confidence intervals for the correlation of metabolites with weight discordance in sIUGR umbilical cord plasma. The left column indicates the between-twin pair regression analysis based on average twin pair birth weights, while the right column indicates the within-twin pair regression analysis based on pair differences in birth weight. The center dotted line in each column indicates 0 correlation; metabolites to the right of the dotted line are positively correlated with weight discordance, whereas metabolites to the left of the dotted line are negatively correlated with weight discordance. In addition, the distance between the metabolites and the dotted line represents the strength of the correlation. Metabolites are classified in accordance with their chemical properties, and only the significant metabolites with p-values less than 0.05 are plotted.

**Figure 5:**
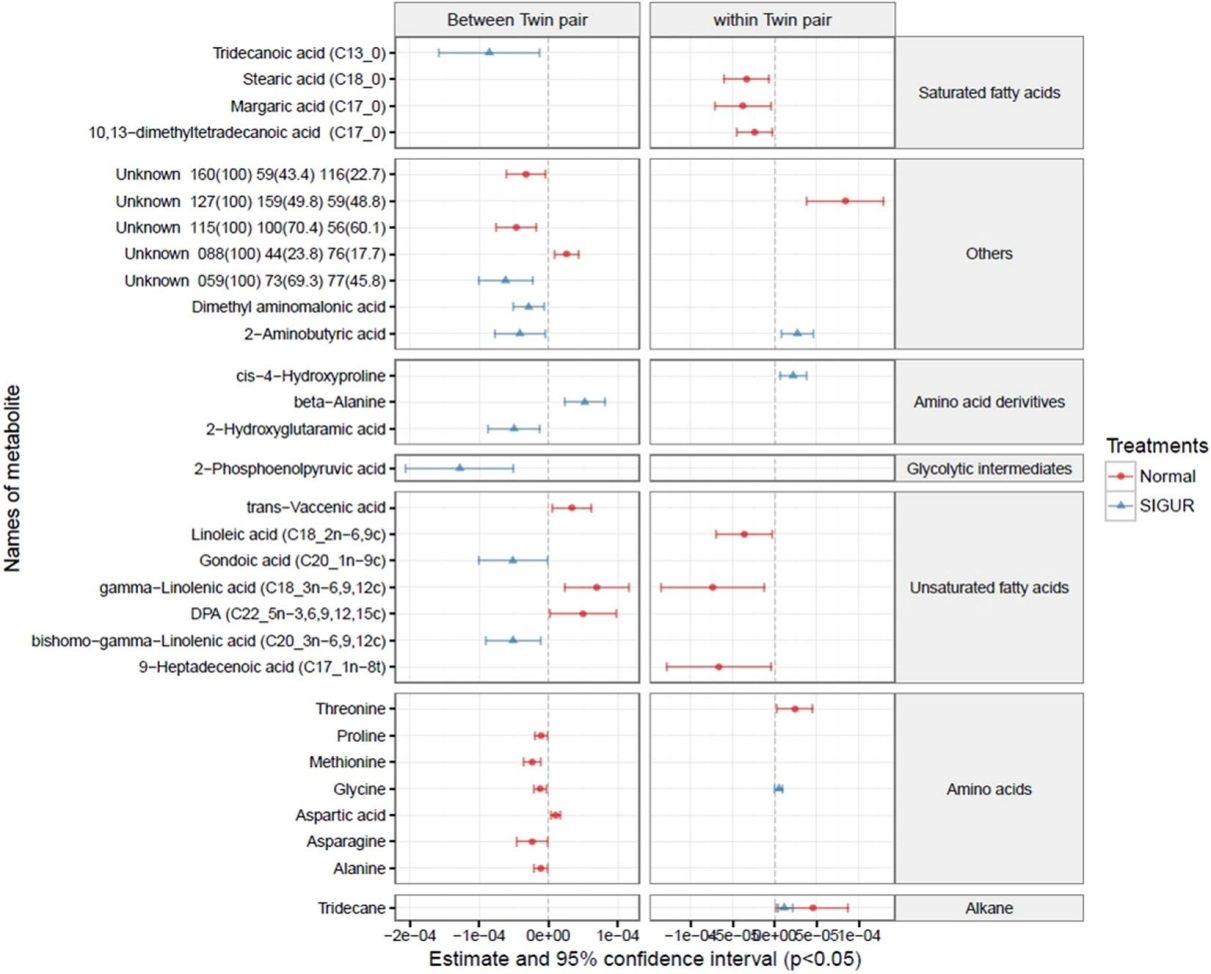
Correlation of birth weight discordance with placenta metabolites within and between twin pairs from sIUGR and normal twins, analyzed using a generalized estimating equation (GEE). The red lines represent the 95% confidence intervals for the correlation of metabolites with weight discordance in the control placenta. The blue lines represent the 95% confidence intervals for the correlation of metabolites with weight discordance in the sIUGR placenta. The left column indicates the between-twin pair regression analysis based on average twin pair birth weights, while the right column indicates the within-twin pair regression analysis based on pair differences in birth weight. The center dotted line in each column indicates 0 correlation; metabolites to the right of the dotted line are positively correlated with weight discordance, whereas metabolites to the left of the dotted line are negatively correlated with weight discordance. In addition, the distance between the metabolites and the dotted line represents the strength of the correlation. Metabolites are classified in accordance with their chemical properties, and only the significant metabolites with p-values less than 0.05 are plotted.

The GEE model separately compares inter (between) and intra (within) twin pairs, thus allowing the isolation of metabolites that are significantly correlated with only birth weight discordance between sIUGR co-twins. From the GEE model, noteworthy metabolites (p ≤ 0.05) that showed correlation with only birth weight discordance from the intra sIUGR co-twin comparison were chosen (the same metabolite shows no correlation with weight discordance from intra and inter control co-twin comparisons). Using these selection criteria, pyroglutamic acid, hexanoic acid, 3-hydroxyoctanoic acid, octadecamethyl cyclononasiloxane, and 1-amniocycopropane-1-carboxylic acid were selected in the umbilical cord blood (Fig. 4). On the other hand, using these same selection criteria, cis-4-hydroxyproline and 2-aminobutyric acid were selected in the placenta (Fig. 5).

### Pairwise comparisons of individual fetuses from sIUGR twins, control twins, and IUGR singletons

Linear logistic regression adjusted for gestational age (confounding factor) identified distinct sets of significant metabolites for the umbilical cord plasma and placental samples (p < 0.05). The results of the 20-pairwise cross comparisons among control twin individuals, sIUGR twin individuals, and IUGR singletons are shown in the supplementary tables.

Comparisons among the sIUGR individuals and control twins revealed five classes of metabolites that were significantly altered in the umbilical cord plasma and placental tissues of sIUGR twins, including amino acids, carboxylic acid, saturated fatty acids, unsaturated fatty acids, and polyunsaturated fatty acids (p < 0.05). Among these metabolites, results similar to the PLS-DA between sIUGR twins and control twins were observed. For example, notable significant cord plasma metabolites included methionine and phenylalanine (Tables S6–S10), and notable significant placental metabolites included pyroglutamic acid and N-alpha-acetyl lysine (Tables S16–S20). Similarly, a number of cyclic siloxane xenobiotic compounds were also identified to be significantly different when comparing individual sIUGRs to individual control twins in both the umbilical cord plasma and placenta (bold labels in Tables S6–S25). These xenobiotics included octadecamethyl cyclononasiloxane, tetradecamethyl cycloheptasiloxane, and cyclopentasiloxane decamethyl. Interestingly, tetradecamethyl cycloheptasiloxane and octadecamethyl cyclononasiloxane were the only metabolites identified as being significantly different in sIUGR co-twins (Table S11).

Furthermore, 16 metabolites in the umbilical cord plasma were significantly different between IUGR singletons and control larger twins (Table S12), while 18 metabolites were significantly different between IUGR singletons and control smaller twins (Table S13). However, only six metabolites were significantly different between the umbilical cord plasma samples from IUGR singletons and sIUGR larger twins (Table S14) as well as between IUGR singletons and sIUGR smaller twins (Table S15). For placental samples, creatinine was the only metabolite significantly different between IUGR singletons and control larger twins (Table S22) as well as between IUGR singletons and control smaller twins (Table S23). Additionally, glycine, trans-vaccenic acid, behenic acid, and adrenic acid were significantly different between IUGR singletons and sIUGR larger twins (Table S24), whereas 11 metabolites significantly differed between IUGR singletons and sIUGR smaller twins (Table S25). Collectively, significant differences in the amino acid composition of the umbilical cord plasma were found between IUGR singletons and control twins, whereas significant differences were observed in the fatty acid compositions of both umbilical cord plasma and placenta samples between IUGR singletons and sIUGR twins.

## Discussion

Intricate alterations or imbalances of feto-maternal interactions during pregnancy lead to adverse birth outcomes or subsequent health issues for the mother and offspring later in life. Currently, very few untargeted metabolomic studies on abnormal fetal growth have been reported^20–23^; only one study examined the metabolic profile of the sIUGR subtype for the specific purpose of identifying metabolites related to endothelial damage, while the others studied singleton IUGR pregnancies. Therefore, to the best of our knowledge, the present study is the first to comprehensively investigate the umbilical cord blood and placental metabolomes of sIUGR twins^20–23^.

We found that the umbilical cord plasma metabolome allowed better discrimination of sIUGR twins from control twins and IUGR singletons than that of the placenta. Hellmuth *et al*.^24^ also showed an association of the umbilical cord blood metabolome with infant birthweight. In monochorionic twins, the co-twins share the same placenta, but cord blood is transported via a randomly inserted umbilical cord to independently acquire the essential amino acids that are vital for fetal development and growth^25^. In addition, umbilical cord blood holds key information that can reflect fetal metabolism, maternal metabolism, and fetal-maternal transport via the placenta^24^.

Among the significantly differentiated metabolites between sIUGR twins and control twins, methionine is quite attractive. It is an essential amino acid critical for protein synthesis and a key source of methyl groups for methylation reactions that affect several biochemical pathways involved in the production of nutrients crucial for the optimal functioning of the cardiovascular, skeletal, and nervous systems^26,27^. Previous research by Horgan *et al*. identified the metabolic disruption produced in placental explants by oxygen deprivation, during which methionine is upregulated^21^. The elevation of methionine was also identified in the umbilical cord of singleton IUGRs^22^. Collectively, these studies suggest that the methionine-cysteine metabolism involving the intermediate homocysteine may be altered in IUGR. Hyperhomocysteinemia, a result from this alteration of methionine-cysteine metabolism, is associated with growth restriction^28^. Our pathway analysis results have also confirmed the disruption of the methionine-cysteine metabolism in sIUGR twins.

In addition to methionine, phenylalanine, which must be obtained from a dietary source in humans, is another essential amino acid significantly elevated in the umbilical cord blood of sIUGR twins. One of the pathways involving phenylalanine in the human body is its conversion to tyrosine via phenylalanine hydroxylase and its incorporation into the monoamine neurotransmitters dopamine, norepinephrine (noradrenaline), and epinephrine (adrenaline). Our results also revealed significantly higher levels of tyrosine in the sIUGR twins, and our pathway analysis pointed towards the disruption of both the phenylalanine and tyrosine biochemical pathways in sIUGR twins. However, this finding is controversial among various studies. For example, phenylalanine amounts have been found to be lower in umbilical venous samples from singleton IUGR and sIUGR twins^29,30^. On the other hand, the results from our study agree with those of Cosmi *et al*.^20^, who identified a trend for increased phenylalanine in the umbilical cord blood of sIUGR twins with Doppler velocimetry alterations. Favretto *et al*.^22^ also reported an increase in the concentration of phenylalanine in the umbilical cord blood of singleton fetuses with IUGR. Therefore, phenylalanine levels and their association with fetal growth restriction are worthy of further investigation. Buildup of phenylalanine in the body is possibly due to phenylketonuria (PKU), an inherited disease in which the phenylalanine hydroxylase gene is mutated^31^. Maternal PKU (pregnant women with existing PKU) is known to result in lower neonatal birthweight^32^.

Interestingly, we observed elevated levels of N-alpha-acetyl lysine (a lysine derivative) in the placentas of sIUGR twins compared to that in control twins. The acetylation of lysine residues is an important epigenetic modification that regulates gene expression^33^. Animal studies have shown that lysine derivatives are related to epigenetic changes associated with not only IUGR but also with uteroplacental insufficiency^34^. However, the involvement of lysine derivatives and their association with epigenetic regulation in the development of sIUGR requires further mechanistic validation.

Pyroglutamic acid, also known as 5-oxoproline, was found to be elevated in the placentas of sIUGR twins, and glutamic acid levels were significantly lower in these twins compared to those in control twins. To our knowledge, one study by Dess *et al*. (2016) showed an opposite relationship between pyroglutamic acid and fetal growth, in contrast to our findings. As shown by Dess, pyroglutamic acid in the sIUGR offspring after birth was upregulated due to breast milk feeding^35^. This indirectly signifies that a lack of pyroglutamic acid may exert an undesirable effect on fetal growth and development. A minimal number of studies investigating links between glutamic acid and fetal growth have been published. Pyroglutamic acid and glutamic acid are key intermediates in glutathione metabolism, and the results of our pathway analysis clearly indicated a possible disruption of the glutathione biochemical pathway in the placentas of sIUGR twins. Therefore, the mechanistic function and role of glutathione metabolism in intrauterine growth clearly warrants further investigation.

Twins provide an excellent opportunity to gain a better understanding of intrauterine and extrauterine factors that may be directly associated with the phenotypic discordance between sIUGR co-twins. Twins are of special interest because they provide naturally matched pairs in which the confounding effects of a large number of potentially causal factors (such as maternal nutrition and length of gestation) can be removed by comparisons between twins who share them. We compared the metabolic profiles of sIUGR larger twins and sIUGR smaller twins to understand the potential relationship between sIUGR co-twins and birthweight discordance. MCDA twins were chosen for this study because they share not only a maternal intrauterine environment and placenta but also identical genetics. Nevertheless, there are obvious differences in sIUGR twin birthweight phenotypes. The study of placental pathologies, especially anatomical factors, such as the location of the two umbilical cords randomly inserted into the placenta, interstitial vascular anastomoses within co-twins, and highly diverse lesions, has always been the classical direction of sIUGR research^36^. These anatomical factors result in imbalance between the placental local blood flow and a consequential discordance in nutrient supply between the two fetuses. Apart from placental anatomical reasons, we aimed to explain the imbalanced nutrients supplied to MCDA twins caused by unequal placental blood flow at the molecular level.

One interesting finding from this work was the existence of xenobiotics in the umbilical cord blood and placentas of sIUGR twins and control twins. Selective xenobiotic levels were significantly higher in the umbilical cord plasma samples of the smaller sIUGR co-twin fetuses and significantly higher in sIUGR twins compared to that in normal twins. The xenobiotics of interest were identified as cyclic siloxanes, including cyclononasiloxane octadecamethyl, cycloheptasiloxane tetradecamethyl, and cyclopentasiloxane decamethyl. This finding indicates that despite the placenta functioning as the barrier to the fetal-maternal interface, xenobiotics (<1 kDa) taken up by the mother could readily cross into fetal circulation^37^. The xenobiotics identified in this study, including poly-aromatic hydrocarbons, organophosphate pesticides, and chlorinated compounds, have previously been found to result in low birth weight^38^. Cyclic siloxanes are mostly found in fuel additives, personal care products, and biomedical devices. Limited evidence suggests that cyclic siloxanes may be toxic for laboratory animals and can impair fertility and reproduction^39–42^. Our study is the first report of an association between cyclic siloxanes and sIUGR. Although the underlying mechanisms of cyclic siloxanes in regulating fetal development remains largely unknown, its appearance in the umbilical cord blood and placenta highlights the possibility of extrauterine environmental factors leading to weight discordance between twins sharing identical genetics.

GEE was applied in this study to remove inter-twin pair confounding effects from a large number of potentially causal factors (such as maternal nutrition and length of gestation)^43^. Our results revealed that three types of metabolites might contribute to weight discordance between sIUGR co-twins: pyroglutamic acid, cis-4-hydroxyproline, and cyclic siloxanes. The enrichment of cis-4-hydroxyproline in the placenta was positively correlated with birth weight discordance between sIUGR co-twins. Previous studies have shown that cis-4-hyrdoxyproline is a proline analog that inhibits hydroxyproline synthesis, which plays a critical role in type V collagen formation, thus affecting placental structure, stability, and formation^44,45^. This finding may result in pregnancy-related complications, such as IUGR and preeclampsia^46,47^. Our second finding suggests that as the sIUGR weight discordance increases, the pyroglutamic acid concentration in the umbilical cord plasma increases. The significance of pyroglutamic acid has been discussed previously. The xenobiotic compound octadecamethyl cyclononasiloxane is another potential contributor to sIUGR weight discordance. This finding also supports the discovery from our previous analysis that cyclic siloxane levels were higher in sIUGR twins than in control twins, thus providing further evidence of the effect of environmental factors on fetal development.

Our study was limited by the complexity of the sIUGR twin model and high FDRs for a few findings. Despite that non-sIUGR twin pairs may also have smaller birth weight discordance, we have confirmed that all control twin pairs have an intrauterine fetal size discordance less than 20% at birth. Another limitation of this study is that it did not include the appropriate for gestational age (AGA) singletons as a reference group for IUGR singletons. Although this comparison makes the investigation of singletons more comprehensive, the main objective of this study was to compare sIUGR in MCDA twins. Performing this additional AGA analysis would have made the statistical analysis even more complex and deviated from our main theme. However, this study successfully analyzed the comprehensive metabolite profiles of the umbilical cord blood and placenta, which has not previously been reported. Our work provides a starting point for future targeted investigations of the metabolome in the birthweight discordance observed in sIUGR.

## Methods

### Study population

**Figure 6:**
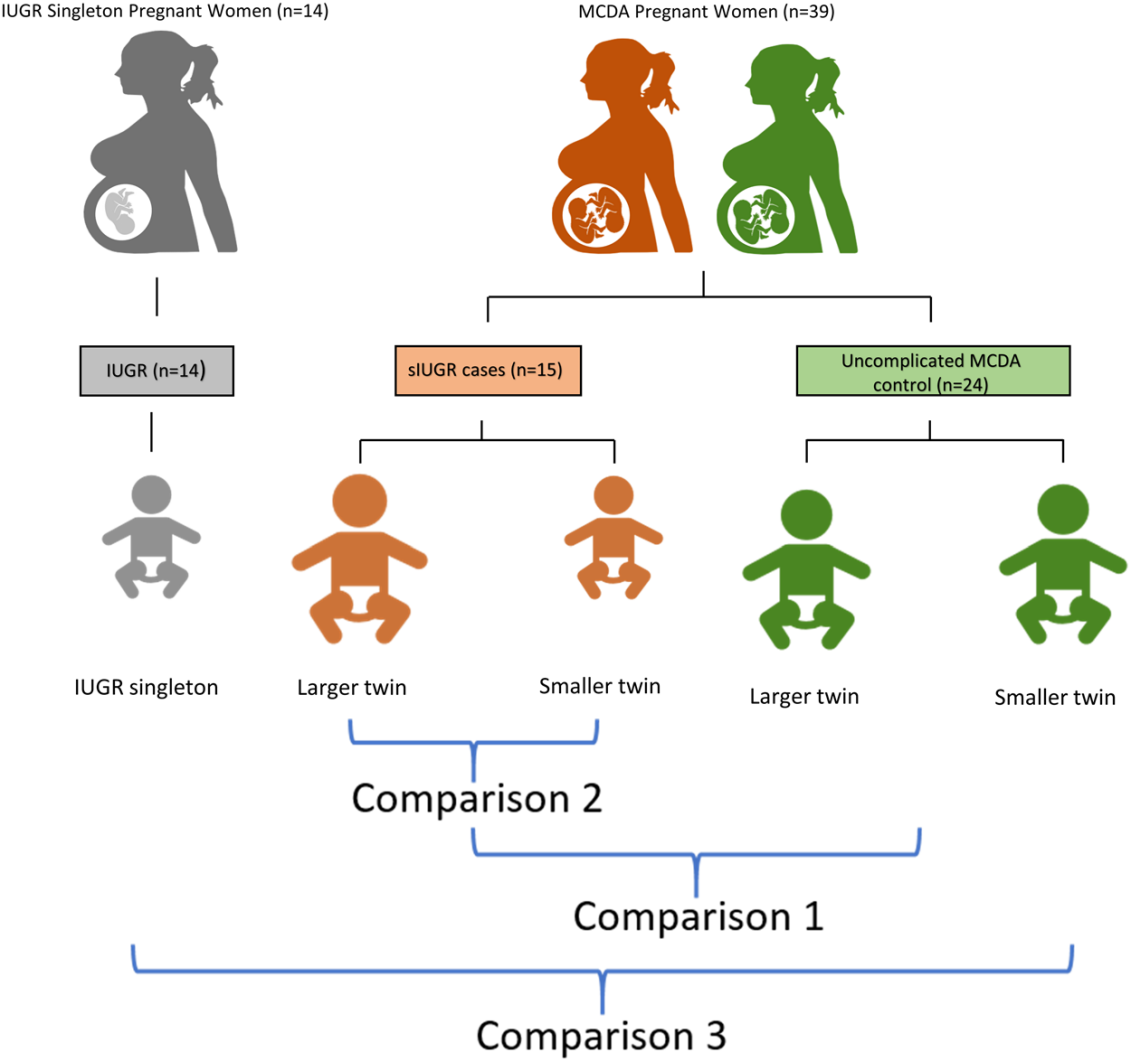
Overall study design. Thirty-nine women with an MCDA pregnancy and 14 singleton IUGR pregnancies were selected. Among the twin births, 15 were identified as having sIUGR, while the other 24 were uncomplicated twin pregnancies. The single fetus group had intrauterine growth restriction (IUGR). Umbilical cord blood and placental tissue samples were collected from the mothers during delivery and analyzed using GC-MS based metabolomics. Comparison 1 investigates the metabolic differences between control twins and sIUGR twins. Comparison 2 explores the phenotypic disparity that exists between sIUGR larger twins and sIUGR smaller twins. Comparison 3 compares IUGR singletons, sIUGR twins, and control twins.

Fifteen MCDA twin pregnancies diagnosed with sIUGR, 24 uncomplicated MCDA twins pregnancies (control), and 14 singleton pregnancies diagnosed with IUGR (Fig. 6) were recruited from the Department of Obstetrics at The First Affiliated Hospital of Chongqing Medical University. All the twin pregnancies were monochorionic twins, were identified by ultrasound and were recruited for research at the gestational age of 12 weeks followed by routine ultrasound examination every two weeks post-recruitment. The chorionicity of the twin pregnancies was further confirmed by experienced obstricians and widwives during delivery^48^. In addition, twin-to-twin transfusion syndrome (TTTS) was ruled-out by the following internationally recognized ultrasound criteria: a monochorionic twin pregnancy with polyhydramnios (deepest amniotic sac being 8 cm in the larger twin pairs and 2 cm in the smaller twin pairs)^49^. In this study, sIUGR was defined by either (1) the birth weight of one twin being less than the 10^th^ percentile at the gestational age or (2) the presence of significant intrauterine fetal size discordance (difference in estimated fetal weight (EFW) of greater than 20%)^15^. IUGR was defined as a birth weight less than the 10^th^ percentile at the gestational age compared to the normal birth weight^50^. The control MCDA twin group had appropriate fetal development, a discordance in fetal birth weight of less than 10%, appropriate amniotic fluid volumes, normal Doppler velocimetry in the umbilical arteries, and similar measurements of the middle cerebral artery-peak systolic velocity among both twins^49^. All pregnancies in this study were delivered by caesarean section. This study was in accordance with the principles established in the Declaration of Helsinki and approved by the Ethical Committee of Chongqing Medical University (201530). Informed consent was obtained from each participant.

### Sample collection

Placental tissue and umbilical cord plasma samples were obtained from participants immediately after delivery. The region of placental tissue sampled was in close proximity to the insertion point for each umbilical cord. A 5 mm^3^ piece of tissue was removed from the fetal side of the placenta, immediately washed in pre-cooled saline solution to remove blood clots, and then blotted dry and frozen in liquid nitrogen until further use. A total of 4 mL of blood was collected from each of the umbilical veins into EDTA-coated blood collection tubes, which were then centrifuged twice at 3,000 rpm for 10 min at 4 °C. The supernatant plasma was aliquoted, transferred into cryopreservation tubes (Micronic, Holland), and stored at −80 °C until further use.

### Metabolite Extraction from Placental Tissue and Cord Plasma

Cold methanol/water (1:1 v/v) coupled with sonication (repeated 5 times for 10 seconds) was used to extract metabolites from isolated placental tissues. In total, 150 µl of plasma sample was mixed with 510 µl of pre-chilled methanol and then frozen at −20 °C for 30 min to precipitate the protein. The internal standard 2,3,3,3-d4-alanine (0.3 μmol) was added to every specimen before extraction. Both plasma and placental supernatants were isolated after centrifugation at 12,000 rpm for 15 min at 4 °C. The supernatants were then dehydrated in a Speed Vac (Labconco, USA) at room temperature for 7 hrs. Dried supernatants were kept at −80 °C prior to chemical derivatization.

### Methyl Chloroform Derivatization and Gas Chromatography-Mass Spectrometry (GC-MS) Analysis

The extracted samples were chemically derivatized via the methyl chloroformate (MCF) method in accordance with the protocol published by Smart *et al*.^51^. MCF-derivatized compounds were examined by an Agilent GC7890B system coupled to a MSD5977A mass selective detector with the electron impact voltage set to 70 eV. The GC column used for metabolite separation was the ZB-1701 GC capillary column (30 m × 250 μm id × 0.15 μm with a 5 m guard column, Phenomenex). The GC temperature was programmed according to the recommendations of Smart *et al*.^51^.

### GC-MS Data Mining and Data Normalization

The automated Mass Spectral Deconvolution and Identification System (AMDIS)^52^ was implemented to deconvolute overlapping chromatograms. Metabolite identifications were conducted using the in-house MCF mass spectral library established by Silas Villas Boas’s metabolomics laboratory in New Zealand^51^. The metabolite identifications were determined based on their spectra match to the mass spectral library and correct chromatographic retention time. The relative concentrations of the identified metabolites were determined using modified XCMS^53^ scripts that selected the peak height of a chosen reference ion within a correct retention time window. The metabolite levels were then normalized by the relative level of the internal standard (2,3,3,3-d4-alanine) in the corresponding sample. Batch variation was removed using median centering based on the control samples.

### Statistical analyses

#### Clinical Statistics

To investigate prenatal clinical characteristics among the pregnant subjects, Student’s *t*-test was used to compare normally distributed data, which included the maternal body mass index (BMI), maternal age, and birth weight. The non-parametric Mann-Whitney U test was used to compare non-normally distributed data consisting of the gestational age, placenta weight, placenta volume, birth weight discordance, amniotic fluid volume (AFV), and Apgar score. For categorical variables, such as the neonatal sex and primigravida, a Chi-square test was applied, and Fisher’s exact test was used to compare the methods of delivery and smoking status.

#### Metabolomics statistical analysis

Univariate Student’s *t*-tests were performed using the Metaboanalyst 3.0 package for R^54^ to screen for significant metabolites and detect metabolic profile differences between the sIUGR twins and control twins. Partial least squares discriminant analysis (PLS-DA) was also used as a supervised multivariate classification method to compare sIUGR twins and control twins. The PLS-DA model was validated using permutation, with the model’s performance assessed by accuracy during training prediction, measured as a p-value. Important classifier metabolites were also identified from the PLS-DA model and ranked based on their variable importance in projection (VIP) scores. Linear logistic regression comparisons between the sIUGR twins, control twins, and IUGR singletons were performed using the general linear model (glm)^55^ logistic regression package in R to identify significant metabolites (p-value cut off ≤ 0.05). The linear logistic regression models were also adjusted to account for gestational age as a potential confounding factor. Generalized estimating equation modeling (GEE) was performed to identify metabolites correlated with weight discordances between sIUGR twins and control twins. The constructed GEE models accommodate within-twin pair and between-twin pair comparisons to rank significantly weighted discordance-correlated metabolites (p-value cut off ≤ 0.05). Exploratory statistics, including quantitative enrichment analysis (QEA)^56^, were performed using MetaboAnalyst 3.0 for metabolite pathway analysis (referencing KEGG)^57^ to investigate functional differences between the control twins and sIUGR twins.

## Electronic supplementary material

Supplementary information

## Data Availability

Statement Supporting data and essential materials for reproducibility of this study are available upon request made to the corresponding authors.
