# Supplementary material for: Metabolic Biomarkers of Monochorionic Twins Complicated With Selective Intrauterine Growth Restriction in Cord Plasma and Placental Tissue
Source: Sci Rep. 2018 Oct 29;8:15914. doi: 10.1038/s41598-018-33788-y (PMC6206027; doi:10.1038/s41598-018-33788-y)
Supplement: Supplementary file 1 — Supplementary information [file 41598_2018_33788_MOESM1_ESM.docx]

**Metabolic Biomarkers of Monochorionic Twins Complicated With Selective Intrauterine Growth Restriction in Cord Plasma and Placental Tissue**

Lianlian Wang^1,2,3,4^, Ting-Li Han^1,2,3,5^, Xiaofang Luo^1,2,3^, Siming Li^6^, Tim Young^6^, Chang Chen^1,2,7^, Li Wen^1,2,3^, Ping Xu^1,2,3^, Yangxi Zheng^1,2,3^, Richard Saffery^8^, Philip N. Baker^1,2,5,9^, Chao Tong^1,2,3*^, Hongbo Qi^1,2,3*^

**Supplementary Information**

**Table S1. Histopathological assessments of placenta.**

| **Group** | **Placental weight (g)** | **Placental length (cm)** | **Placental width (cm)** | **Placental height (cm)** | **Placental volume**  **(cm^3^)** | **Placental histopathological diagnosis*** | | **Color Doppler assessment of placenta**** |
| --- | --- | --- | --- | --- | --- | --- | --- | --- |
| **sIUGR** |  |  |  |  |  |  |  | |
| Sample 1 | 700 | 17 | 18 | 3 | 918 | Normal | Normal | |
| Sample 2 | 550 | 18 | 17 | 2 | 612 | Abnormal^1^ | Normal | |
| Sample 3 | 550 | 20 | 18 | 2.5 | 900 | Abnormal^1^ | Normal | |
| Sample 4 | 550 | 23 | 20 | 2 | 920 | Abnormal^1^ | Normal | |
| Sample 5 | 650 | 20 | 18 | 3 | 1080 | Normal | Normal | |
| Sample 6 | 600 | 19 | 20 | 2 | 760 | Normal | Normal | |
| Sample 7 | 810 | 25 | 21 | 2.5 | 1312.5 | Abnormal^1^ | Normal | |
| Sample 8 | 600 | 20 | 19 | 1.8 | 684 | Normal | Normal | |
| Sample 9 | 800 | 20 | 18 | 2 | 720 | Abnormal^1^ | Normal | |
| Sample 10 | 600 | 22 | 20 | 3 | 1320 | Normal | Normal | |
| Sample 11 | 810 | 18 | 17 | 2 | 612 | Normal | Normal | |
| Sample 12 | 600 | 22 | 20 | 3 | 1320 | Normal | Normal | |
| Sample 13 | 800 | 22 | 18 | 3 | 1188 | Normal | Normal | |
| Sample 14 | 630 | 25 | 23 | 2.5 | 1437.5 | Normal | Normal | |
| Sample 15 | 650 | 22 | 21 | 2 | 924 | Normal | Normal | |
| **Normal** |  |  |  |  |  |  |  | |
| Sample 1 | 720 | 25 | 24 | 3 | 1800 | Normal | Normal | |
| Sample 2 | 790 | 24 | 23 | 2 | 1104 | Normal | Normal | |
| Sample 3 | 670 | 18 | 18 | 4 | 1296 | Normal | Normal | |
| Sample 4 | 600 | 22 | 20 | 3 | 1320 | Normal | Normal | |
| Sample 5 | 700 | 25 | 25 | 2.5 | 1562.5 | Normal | Normal | |
| Sample 6 | 550 | 16 | 18 | 2 | 576 | Normal | Normal | |
| Sample 7 | 600 | 18 | 17 | 2 | 612 | Normal | Normal | |
| Sample 8 | 900 | 22 | 24 | 6 | 3168 | Normal | Normal | |
| Sample 9 | 870 | 35 | 23 | 2 | 1610 | Normal | Normal | |
| Sample 10 | 700 | 33 | 31 | 2 | 2046 | Normal | Normal | |
| Sample 11 | 840 | 22 | 21 | 3 | 1386 | Normal | Normal | |
| Sample 12 | 950 | 26 | 24 | 3 | 1872 | Normal | Normal | |
| Sample 13 | 600 | 29 | 26 | 2.5 | 1885 | Normal | Normal | |
| Sample 14 | 750 | 30 | 24 | 2 | 1440 | Normal | Normal | |
| Sample 15 | 1200 | 35 | 32 | 2 | 2240 | Normal | Normal | |
| Sample 16 | 900 | 23 | 22 | 3 | 1518 | Normal | Normal | |
| Sample 17 | 1020 | 20 | 19 | 2.5 | 950 | Normal | Normal | |
| Sample 18 | 700 | 22 | 20 | 2 | 880 | Normal | Normal | |
| Sample 19 | 650 | 24 | 27 | 2.5 | 1620 | Normal | Normal | |
| Sample 20 | 760 | 30 | 21 | 2 | 1260 | Normal | Normal | |
| Sample 21 | 1180 | 24 | 25 | 2.5 | 1500 | Normal | Normal | |
| Sample 22 | 1250 | 19 | 17 | 3 | 969 | Normal | Normal | |
| Sample 23 | 1230 | 24 | 25 | 2.5 | 1500 | Normal | Normal | |
| Sample 24 | 630 | 19 | 17 | 3 | 969 | Normal | Normal | |

* **Placental histopathological diagnosis**: Routine diagnosis of marginalisation of the umbilical cord’s insertion site^1^, infarctions^2^ or others^3^ by obstritrian at delivery.

** **Color Doppler assessment of placenta**: Determination of placental abnormities such as vascular anastomoses and calcification by Color Doppler at gestational age of 16-18 weeks.

**Table S2. Estimated fetal weight in different stage of pregnancy and birth weight of twins.**

| Group | GA | EFW-L | EFW-S | GA | EFW-L | EFW-S | GA | EFW-L | EFW-S | GA at delivery | BW-L | BW-S |
| --- | --- | --- | --- | --- | --- | --- | --- | --- | --- | --- | --- | --- |
| sIUGR |  |  |  |  |  |  |  |  |  |  |  |  |
| Sample 1 | 16 | 176 | 134 | 24 | 626 | 495 | 32 | 2130 | 1587 | 34.5 | 2560 | 1990 |
| Sample 2 | 18 | 223 | 186 | 24 | 610 | 508 | 32 | 2234 | 1552 | 35.5 | 3100 | 2110 |
| Sample 3 | 19 | 302 | 235 | 26 | 902 | 668 | 31 | 1566 | 1120 | 31.2 | 1600 | 1150 |
| Sample 4 | 17 | 206 | 150 | 25 | 708 | 457 | 33 | 2417 | 1680 | 35.3 | 3030 | 1860 |
| Sample 5 | 19 | 315 | 262 | 24 | 694 | 550 | 32 | 2060 | 1654 | 34.5 | 2380 | 1830 |
| Sample 6 | 16 | 158 | 124 | 24 | 654 | 513 | 32 | 2005 | 1557 | 35.8 | 2250 | 1780 |
| Sample 7 | 16 | 144 | 93 | 24 | 623 | 418 | 31 | 1565 | 1239 | 33 | 1880 | 1130 |
| Sample 8 | 16 | 173 | 131 | 24 | 613 | 419 | 31 | 1704 | 1357 | 36.5 | 2960 | 2210 |
| Sample 9 | 16 | 150 | 142 | 23 | 570 | 512 | 32 | 1885 | 1301 | 33 | 1980 | 1420 |
| Sample 10 | 17 | 182 | 156 | 23 | 532 | 457 | 31 | 1516 | 1158 | 36.7 | 2380 | 1890 |
| Sample 11 | 16 | 148 | 120 | 24 | 644 | 531 | 32 | 2065 | 1634 | 37.7 | 2920 | 2290 |
| Sample 12 | 16 | 152 | 129 | 24 | 745 | 630 | 31 | 1745 | 1334 | 37.3 | 2800 | 2240 |
| Sample 13 | 19 | 310 | 233 | 26 | 1113 | 865 | 31 | 1690 | 1272 | 37.5 | 2840 | 2370 |
| Sample 14 | 18 | 220 | 178 | 24 | 673 | 524 | 32 | 1942 | 1545 | 37.5 | 2780 | 2210 |
| Sample 15 | 18 | 227 | 209 | 24 | 629 | 523 | 31 | 1788 | 1391 | 37.2 | 2940 | 2320 |
| Normal |  |  |  |  |  |  |  |  |  |  |  |  |
| Sample 1 | 18 | 195 | 182 | 24 | 600 | 592 | 32 | 1660 | 1592 | 36.7 | 2500 | 2360 |
| Sample 2 | 18 | 263 | 250 | 23 | 533 | 505 | 32 | 1917 | 1774 | 34.2 | 2170 | 2140 |
| Sample 3 | 16 | 174 | 172 | 24 | 545 | 526 | 33 | 1726 | 1704 | 37.2 | 2450 | 2250 |
| Sample 4 | 17 | 185 | 182 | 25 | 749 | 735 | 33 | 1976 | 1886 | 37.5 | 2670 | 2540 |
| Sample 5 | 19 | 329 | 290 | 25 | 836 | 833 | 33 | 1985 | 1959 | 36.7 | 2510 | 2480 |
| Sample 6 | 18 | 281 | 281 | 24 | 734 | 725 | 33 | 2252 | 2107 | 37.3 | 3060 | 2670 |
| Sample 7 | 18 | 205 | 180 | 25 | 816 | 774 | 32 | 1822 | 1704 | 37.3 | 2770 | 2530 |
| Sample 8 | 16 | 184 | 175 | 24 | 831 | 760 | 32 | 2012 | 1968 | 38.5 | 3270 | 3040 |
| Sample 9 | 19 | 317 | 271 | 24 | 661 | 633 | 33 | 2136 | 2101 | 37.7 | 3100 | 3000 |
| Sample 10 | 16 | 173 | 136 | 24 | 639 | 626 | 33 | 2221 | 2080 | 34.7 | 2300 | 2200 |
| Sample 11 | 17 | 209 | 197 | 24 | 705 | 625 | 33 | 1853 | 1747 | 36 | 2630 | 2380 |
| Sample 12 | 16 | 147 | 145 | 23 | 679 | 614 | 31 | 1952 | 1785 | 35.8 | 2440 | 2220 |
| Sample 13 | 17 | 202 | 201 | 23 | 597 | 585 | 31 | 1802 | 1777 | 38.5 | 3100 | 2700 |
| Sample 14 | 19 | 351 | 346 | 23 | 657 | 642 | 31 | 1949 | 1693 | 35.8 | 2840 | 2760 |
| Sample 15 | 17 | 191 | 189 | 25 | 849 | 820 | 32 | 2015 | 2221 | 37 | 3070 | 2900 |
| Sample 16 | 16 | 179 | 168 | 24 | 708 | 662 | 32 | 1797 | 1752 | 37.8 | 2440 | 2380 |
| Sample 17 | 16 | 170 | 167 | 24 | 725 | 709 | 32 | 2100 | 2057 | 36.5 | 2900 | 2860 |
| Sample 18 | 17 | 200 | 192 | 24 | 678 | 654 | 33 | 2056 | 1940 | 34 | 2500 | 2360 |
| Sample 19 | 16 | 171 | 156 | 24 | 618 | 616 | 33 | 2020 | 1969 | 37.3 | 2700 | 2510 |
| Sample 20 | 17 | 135 | 126 | 24 | 587 | 568 | 33 | 1920 | 1756 | 35 | 2070 | 1910 |
| Sample 21 | 18 | 206 | 189 | 24 | 685 | 606 | 34 | 2198 | 2058 | 37.3 | 2950 | 2900 |
| Sample 22 | 18 | 222 | 201 | 24 | 661 | 624 | 30 | 1970 | 1859 | 34.5 | 2680 | 2680 |
| Sample 23 | 16 | 132 | 105 | 24 | 637 | 602 | 32 | 1767 | 1755 | 35.3 | 2600 | 2430 |
| Sample 24 | 18 | 1891 | 187 | 23 | 478 | 457 | 31 | 1435 | 1403 | 37.2 | 2490 | 2240 |

Abbreviations: GA, gestational age; EFW-L, estimated fetal weight-larger twin; EFW-S, estimated fetal weight-smaller twin; BW-L, birth weight-larger twin; BW-S, birth weight-smaller twin.

**Table S3. Curated metabolites**

| Plasma | Placenta |
| --- | --- |
| 10,13-dimethyltetradecanoic | 10,13-dimethyltetradecanoic acid |
| 11,14-Eicosadienoic | 11,14-Eicosadienoic |
| 1-Aminocyclopropane-1-carboxylic acid | Dimethyl aminomalonic acid |
| 2,4-Di-tert-butylphenol | N-(Carboxymethyl)-L-alanine |
| 2-Aminobutyric acid | 1-Aminocyclopropane-1-carboxylic acid |
| 2-Hydroxybutyric acid | 2-Aminoadipic acid |
| 3-(-2-Thienyl)-D-alanine | 2-Aminobutyric acid |
| 3-Hydroxydecanoic acid | 2-Hydroxybutyric acid |
| 3-Hydroxyoctanoic acid | 2-Hydroxyglutaramic acid |
| 3-Methyl-2-oxopentanoic acid | 2-Methyloctadecanoic acid |
| 4-Aminobutyric acid | 2-Oxoglutaric acid |
| 4-Hydroxyphenylacetic acid | 2-Phosphoenolpyruvic acid |
| 4-Methyl-2-oxopentanoic acid | 4-Aminobutyric acid |
| 9E-Heptadecenoic acid | 9E-Heptadecenoic acid |
| Adipic acid | Adipic acid |
| Adrenic acid | Adrenic acid |
| Alanine | Alanine |
| Arachidonic acid | Arachidic acid |
| Asparagine | Arachidonic acid |
| Azelaic acid | Asparagine |
| Benzoic acid | Aspartic acid |
| beta-Alanine | Behenic acid |
| bishomo-gamma-Linolenic acid | Benzoic acid |
| Caffeine | beta-Alanine |
| Caprylic acid | beta-Citryl-L-glutamic acid |
| cis-4-Hydroxyproline | bishomo-gamma-Linolenic acid |
| cis-Aconitic acid | Caprinoic acid |
| Citric acid | Caprylic acid |
| Creatinine | cis-4-Hydroxyproline |
| Cysteine | cis-Aconitic acid |
| Docosahexaenoic acid | Citric acid |
| DL-3-Aminoisobutyric acid | Creatinine |
| DL-gamma-methyl-ketoglutaramate | Cysteine |
| Dodecane | Dehydroascorbic acid |
| Dodecanoic acid | Docosahexaenoic acid |
| Eicosapentaenoic acid | DL-3-Aminoisobutyric acid |
| Fumaric acid | Dodecane |
| gamma-Linolenic acid | Dodecanoic acid |
| Glutamic acid | Docosapentaenoic acid |
| Glutamine | Fumaric acid |
| Glutaric acid | gamma-Linolenic acid |
| Glutathione | Glutamic acid |
| Glyceric acid | Glutamine |
| Glycine | Glutaric acid |
| Glyoxylic acid | Glutathione |
| Hexanoic acid | Glyceric acid |
| Hippuric acid | Glycine |
| Histidine | Glyoxylic acid |
| Isoleucine | Gondoic acid |
| Itaconic acid | Hexanoic acid |
| Lactic acid | Histidine |
| Leucine | Hydroxybenzoic acid |
| Linoleic acid | Isoleucine |
| Lysine | Itaconic acid |
| Malic acid | Lactic acid |
| Margaric acid | Leucine |
| Methionine | Linoleic acid |
| Myristic acid | Lysine |
| Myristoleic acid | Malic acid |
| Nicotinamide | Malonic acid |
| Nonadecanoic acid | Margaric acid |
| Ornithine | Methionine |
| Oxalic acid | Myristic acid |
| Palmitelaidic acid | N-alpha-Acetyllysine |
| Pentadecanoic acid | Nicotinamide |
| Phenylalanine | Nicotinic acid |
| Proline | Nonadecanoic acid |
| Pyroglutamic acid | Ornithine |
| Pyruvic acid | Oxalic acid |
| Serine | Palmitelaidic acid |
| Stearic acid | Pentadecanoic acid |
| Succinic acid | Phenylalanine |
| Threonine | Proline |
| trans-4-Hydroxyproline | Pyroglutamic acid |
| trans-Vaccenic acid | Pyruvic acid |
| Tridecane | Serine |
| Tryptophan | Stearic acid |
| Tyrosine | Succinic acid |
| Valine | Threonine |
|  | trans-Vaccenic acid |
|  | Tridecane |
|  | Tridecanoic acid |
|  | Tryptophan |
|  | Valine |

**Table S4.** Pathways Identified by the by the targeted pathway and quantitative enrichment analysis (QEA) using MetaboAnalyst 3.0 between the umbilical cord blood of the control twins vs the sIUGR twins

| Pathway name | Match status | *p*-value | -log（p） | FDR | Impact |
| --- | --- | --- | --- | --- | --- |
| Phenylalanine metabolism | 8/45 | 4.8349E-5 | 9.9371 | 0.0024658 | 0.19802 |
| Tyrosine metabolism | 5/76 | 2.704E-4 | 8.2156 | 0.0068953 | 0.10681 |
| Cysteine ​​and methionine metabolism | 7/56 | 8.3985E-4 | 7.0823 | 0.014277 | 0.20887 |
| Phenylalanine, tyrosine and tryptophan biosynthesis | 3/27 | 0.001997 | 6.2161 | 0.025461 | 0.008 |
| Aminoacyl-tRNA biosynthesis | 18/75 | 0.0029465 | 5.8271 | 0.030054 | 0.16902 |
| Nitrogen metabolism | 8/39 | 0.0040007 | 5.5213 | 0.034006 | 0.00763 |
| Ubiquinone and other terpenoids-quinone biosynthesis | 1/36 | 0.0048382 | 5.3312 | 0.03525 | 0.0 |
| Thiamine metabolism | 3/24 | 0.0061267 | 5.0951 | 0.039058 | 0.0 |
| Propionate metabolism | 6/35 | 0.020926 | 3.8668 | 0.11496 | 0.08634 |
| Alanine, Aspartic Acid, and glutamate Metabolism | 8/24 | 0.022541 | 3.7924 | 0.11496 | 0.59164 |
| Butanoate metabolism | 5/40 | 0.029823 | 3.5125 | 0.11779 | 0.13128 |
| D-glutamine and D-glutamate metabolism | 2/11 | 0.030979 | 3.4744 | 0.11779 | 0.13904 |
| Histidine metabolism | 2/44 | 0.032219 | 3.4352 | 0.11779 | 0.14039 |
| Glutathione metabolism | 6/38 | 0.032334 | 3.4316 | 0.11779 | 0.25028 |
| Valine, Leucine and Isoleucine Degradation | 4/40 | 0.052137 | 2.9539 | 0.17178 | 0.06442 |
| Pantothenate acid and CoA biosynthesis | 4/27 | 0.056465 | 2.8741 | 0.17178 | 0.07286 |
| Citrate cycle (TCA cycle) | 6/20 | 0.059123 | 2.8281 | 0.17178 | 0.28353 |
| Taurine and hypotaurine metabolism | 3/20 | 0.062745 | 2.7687 | 0.17178 |  |
| Pyrimidine metabolism | 3/60 | 0.064561 | 2.7401 | 0.17178 | 0.0 |
| Selenoamino acid metabolism | 1/22 | 0.068911 | 2.6749 | 0.17178 | 0.0 |
| Valine, Leucine, Isoleucine biosynthesis | 6/27 | 0.070733 | 2.6488 | 0.17178 | 0.08692 |
| Porphyrin and chlorophyll metabolism | 3/104 | 0.083504 | 2.48829 | 0.18904 | 0.0 |
| Glyoxylate and dicarboxylate metabolism | 8/50 | 0.085728 | 2.4566 | 0.18904 | 0.42071 |
| Arginine and proline metabolism | 10/77 | 0.088961 | 2.4196 | 0.18904 | 0.36417 |
| beta-alanine metabolism | 3/28 | 0.14731 | 1.9152 | 0.30052 | 0.25694 |
| Sulfur metabolism | 2/18 | 0.15903 | 1.8387 | 0.31125 | 0.03307 |
| Lysine biosynthesis | 1/32 | 0.17088 | 1.7668 | 0.31125 | 0.09993 |
| Biotin metabolism | 1/11 | 0.17088 | 1.7668 | 0.31125 | 0.0 |
| Primary bile acid biosynthesis | 1/47 | 0.18455 | 1.6898 | 0.32456 | 0.00822 |
| Glycerolipid metabolites | 1/32 | 0.19847 | 1.6171 | 0.33739 | 0.0206 |
| Lysine degradation | 3/47 | 0.2363 | 1.4427 | 0.38172 | 0.2118 |
| D-arginine and D-ornithine metabolites | 1/8 | 0.2422 | 1.418 | 0.38172 | 0.0 |
| Pentose phosphate pathway | 2/32 | 0.25357 | 1.3721 | 0.38172 | 0.02181 |
| Purine metabolism | 3/92 | 0.25448 | 1.3685 | 0.38172 | 0.0 |
| Nicotinate and nicotinamide metabolism | 3/44 | 0.27342 | 1.2967 | 0.3924 | 0.03827 |
| Caffeine metabolism | 2/21 | 0.30639 | 1.1829 | 0.3924 | 0.18429 |
| Vitamin B6 metabolism | 1/32 | 0.30776 | 1.1784 | 0.3924 | 0.01914 |
| Ascorbate metabolism and aldarate metabolism | 1/45 | 0.30776 | 1.1784 | 0.3924 | 0.01617 |
| Pentose and glucuronate interconversion | 1/53 | 0.30776 | 1.1784 | 0.3924 | 0.0 |
| Terpenoid backbone biosynthesis | 1/33 | 0.30776 | 1.1784 | 0.3924 | 0.0 |
| Fatty acid biosynthesis | 4/49 | 0.31636 | 1.1509 | 0.39352 | 0.0 |
| Glycine, serine and threonine metabolism | 8/48 | 0.32774 | 1.2255 | 0.39797 | 0.42086 |
| Methane metabolism | 2/34 | 0.33772 | 1.0855 | 0.40056 | 0.01751 |
| Cyanamide metabolism | 3/16 | 0.36769 | 1.0005 | 0.42618 | 0.0 |
| Fatty acid metabolism | 1/50 | 0.45697 | 0.78314 | 0.5179 | 0.0 |
| Linoleic acid metabolism | 2/15 | 0.51854 | 0.65673 | 0.56375 | 0.65625 |
| Pyruvate metabolism | 3/32 | 0.53652 | 0.62265 | 0.56375 | 0.3201 |
| Tryptophan metabolism | 1/79 | 0.54083 | 0.61466 | 0.56375 | 0.10853 |
| Arachidonic acid metabolism | 1/62 | 0.54164 | 0.61316 | 0.56375 | 0.21669 |
| Sphingolipid metabolism | 1/25 | 0.56158 | 0.577 | 0.56577 | 0.0 |
| Glycolysis or gluconeogenesis | 2/31 | 0.56577 | 0.56957 | 0.56577 | 0.0953 |

**Table S5.** Pathways Identified by the by the targeted pathway and quantitative enrichment analysis (QEA) using MetaboAnalyst 3.0 between the placenta of the control twins vs the sIUGR twins.

| Pathway name | Match status | *p*-value | -log（p） | FDR | Impact |
| --- | --- | --- | --- | --- | --- |
| Glutathione metabolism | 6/38 | 0.023224 | 3.7626 | 0.69431 | 0.25028 |
| beta-alanine metabolism | 5/28 | 0.04177 | 3.1756 | 0.69431 | 0.26813 |
| Histidine metabolism | 4/44 | 0.04728 | 3.0517 | 0.69431 | 0.14039 |
| Pyrimidine metabolism | 4/60 | 0.056967 | 2.8653 | 0.69431 | 0.0 |
| D-glutamine and D-glutamate metabolism | 3/11 | 0.071359 | 2.64 | 0.69431 | 0.13904 |
| Nicotinate and nicotinamide metabolism | 5/44 | 0.1008 | 2.2946 | 0.69431 | 0.14392 |
| Porphyrin and chlorophyll metabolism | 3/104 | 0.11689 | 2.1465 | 0.69431 | 0.0 |
| Fatty acid metabolism | 1/50 | 0.12717 | 2.0623 | 0.69431 | 0.0 |
| Nitrogen metabolism | 8/39 | 0.1299 | 2.041 | 0.69431 | 0.0083 |
| Alanine, aspartate and glutamate metabolism | 10/24 | 0.14294 | 1.9453 | 0.69431 | 0.8566 |
| Butanoate metabolism | 0.0 | 0.056967 | 2.8653 | 0.58106 | 0.0 |
| Ubiquinone and other terpenoid-quinone biosynthesis | 1/36 | 0.16337 | 1.8118 | 0.69431 | 0.03049 |
| Selenamino acid metabolism | 1/22 | 0.21337 | 1.5447 | 0.83707 | 0.0 |
| Biotin metabolism | 1/11 | 0.24649 | 1.4004 | 0.88896 | 0.0 |
| Cyanoamino acid metabolism | 4/16 | 0.28334 | 0.2611 | 0.88896 | 0.0 |
| Lysine degradation | 4/47 | 0.32848 | 1.1133 | 0.88896 | 0.22743 |
| Aminoacyl-tRNA biosynthesis | 18/75 | 0.34946 | 1.0514 | 0.88896 | 0.22536 |
| Caffeine metabolism | 1/21 | 0.35314 | 1.0409 | 0.88896 | 0.0 |
| Arginine and prolline metabolism | 10/77 | 0.36025 | 1.0209 | 0.88896 | 0.29319 |
| Lysine biosynthesis | 4/32 | 0.4032 | 0.90831 | 0.88896 | 0.16762 |
| Tryptophan metabolism | 1/79 | 0.42454 | 0.85674 | 0.88896 | 0.10853 |
| D-arginine and D-ornithine metabolism | 1/8 | 0.44803 | 0.8029 | 0.88896 | 0.0 |
| Phenylalanine, tyrosine and tryptophan biosynthesis | 3/27 | 0.47134 | 0.75218 | 0.88896 | 6.2E-4 |
| Propanonate metabolism | 5/35 | 0.48581 | 0.72194 | 0.88896 | 0.08634 |
| Glycerollipid metabolism | 1/32 | 0.48803 | 0.71737 | 0.88896 | 0.0206 |
| Pyruvate metabolism | 3/32 | 0.49054 | 0.71225 | 0.88896 | 0.3201 |
| Glycolysis or gluconeogenesis | 3/31 | 0.49054 | 0.71225 | 0.88896 | 0.1988 |
| Tyrosine metabolism | 3/76 | 0.5235 | 0.64721 | 0.88896 | 0.0 |
| Pantothenate and CoA biosynthesis | 5/27 | 0.55037 | 0.59716 | 0.88896 | 0.07286 |
| Phenylalanine metabolism | 6/45 | 0.55346 | 0.59157 | 0.88896 | 0.16652 |
| Sphingolipid metabolism | 1/25 | 0.55437 | 0.58992 | 0.88896 | 0.0 |
| Purine metabolism | 3/92 | 0.5862 | 0.5341 | 0.88896 | 0.0 |
| Taurine and hypotaurine metabolism | 3/20 | 0.63763 | 0.45 | 0.88896 | 0.05395 |
| Primary bile acid biosynthesis | 1/47 | 0.65308 | 0.42605 | 0.88896 | 0.00822 |
| Citric acid cycle | 7/20 | 0.6637 | 0.40993 | 0.88896 | 0.32569 |
| Cysteine ​​and methionine metabolism | 7/56 | 0.67776 | 0.38896 | 0.88896 | 0.20072 |
| Methane metabolism | 2/34 | 0.75022 | 0.2874 | 0.88896 | 0.01751 |
| Arachidonic acid metabolism | 1/62 | 0.77133 | 0.25964 | 0.88896 | 0.21669 |
| Pentose phosphate pathway | 2/32 | 0.77252 | 0.2581 | 0.88896 | 0.02181 |
| Glyoxylate and dicarboxylate metabolism | 8/50 | 0.77299 | 0.25749 | 0.88896 | 0.39651 |
| Linoleic acid metabolism | 2/15 | 0.77847 | 0.25042 | 0.88896 | 0.65625 |
| Glycine, serine and threonine metabolism | 9/48 | 0.79001 | 0.2357 | 0.88896 | 0.42086 |
| Vitamin B6 metabolism | 2/32 | 0.79244 | 0.23264 | 0.88896 | 0.03828 |
| Ascorbate and aldarate metabolism | 2/45 | 0.79244 | 0.23264 | 0.88896 | 0.01617 |
| Sulfur metabolism | 2/18 | 0.79896 | 0.22445 | 0.88896 | 0.03307 |
| Fatty acid biosynthesis | 4/49 | 0.80449 | 0.21754 | 0.88896 | 0.0 |
| Thiamine metabolism | 2/24 | 0.85043 | 0.16201 | 0.88896 | 0.0 |
| Pentose and glucuronate interconversion | 1/53 | 0.8541 | 0.1577 | 0.88896 | 0.0 |
| Terpenoid backbone biosynthesis | 1/33 | 0.8541 | 0.1577 | 0.88896 | 0.0 |
| Valine, leucine and isoleucine Degradation | 3/40 | 0.88151 | 0.12612 | 0.89914 | 0.02232 |
| Valine, leucine and isoleucine Biosynthesis | 5/27 | 0.95622 | 0.04477 | 0.95622 | 0.06148 |

| Table S6 Control vs sIUGR (plasma) | | |
| --- | --- | --- |
| Metabolite | **p value** | **q value** |
| Methionine | 0.001218 | 0.129068 |
| Phenylalanine | 0.004856 | 0.18135 |
| 4-Hydroxyphenylacetic acid | 0.013523 | 0.302543 |
| Cyclopentasiloxane, decamethyl | 0.015322 | 0.302543 |
| 2-Aminobutyric | 0.017125 | 0.302543 |
| Tyrosine | 0.026534 | 0.351752 |
| Eicosapentaenoic acid | 0.026547 | 0.351752 |
| Succinic acid | 0.042616 | 0.439502 |
| Valine | 0.042732 | 0.439502 |
| Adrenic acid | 0.045609 | 0.439502 |

| Table S7 Control L vs sIUGR L (plasma) | | |
| --- | --- | --- |
| Metabolite | **p value** | **q value** |
| Methionine | 0.012623 | 0.879179 |

| Table S8 Control L vs sIUGR S (plasma) | | |
| --- | --- | --- |
| Metabolite | **p value** | **q value** |
| Eicosapentaenoic acid | 0.029666 | 0.966255 |
| Cyclopentasiloxane, decamethyl | 0.044853 | 0.966255 |

| Table S9 Control S vs sIUGR L (plasma) | | |
| --- | --- | --- |
| Metabolite | **p value** | **q value** |
| Methionine | 0.003741 | 0.396544 |
| Phenylalanine | 0.011563 | 0.612829 |
| Adipic acid | 0.045902 | 0.706955 |

| Table S10 Control S vs sIUGR S (plasma) | | |
| --- | --- | --- |
| Metabolite | **p value** | **q value** |
| Methionine | 0.022445 | 0.745079 |
| Cyclopentasiloxane, decamethyl | 0.033493 | 0.745079 |
| Phenylalanine | 0.037687 | 0.745079 |

| Table S11 sIUGR L vs sIUGR S (plasma) | | |
| --- | --- | --- |
| Metabolite | **p value** | **q value** |
| Cycloheptasiloxane, tetradecamethyl | 0.041527 | 0.992026 |
| Cyclononasiloxane, octadecamethyl | 0.04761 | 0.992026 |

| Table S12 IUGR singletons vs Control L (plasma) | | |
| --- | --- | --- |
| Metabolite | **p value** | **q value** |
| Glycine | 0.00986 | 0.174559 |
| Dodecanoic acid | 0.011062 | 0.174559 |
| Phenylalanine | 0.01222 | 0.174559 |
| 2-Aminobutyric acid | 0.012582 | 0.174559 |
| Malic acid | 0.012597 | 0.174559 |
| 4-Hydroxyphenylacetic acid | 0.014873 | 0.180341 |
| 4-Methyl-2-oxopentanoic acid | 0.016892 | 0.182059 |
| gamma-Linolenic | 0.021228 | 0.183013 |
| Methionine | 0.022641 | 0.183013 |
| Arachidonic acid | 0.028763 | 0.18941 |
| Valine | 0.032135 | 0.18941 |
| Stearic acid | 0.034517 | 0.18941 |
| Serine | 0.034972 | 0.18941 |
| Palmitelaidic acid | 0.03631 | 0.18941 |
| Threonine | 0.037101 | 0.18941 |
| Itaconic acid | 0.039744 | 0.192758 |

| Table S13 IUGR singletons vs Control S (plasma) | | |
| --- | --- | --- |
| Metabolite | **p value** | **q value** |
| 2-Aminobutyric acid | 0.007768 | 0.151992 |
| Dodecanoic | 0.008789 | 0.151992 |
| Creatinine | 0.014991 | 0.151992 |
| 4-Aminobutyric acid (GABA) | 0.017559 | 0.151992 |
| Palmitelaidic acid | 0.017889 | 0.151992 |
| 4-Hydroxyphenylacetic acid | 0.018182 | 0.151992 |
| Malic acid | 0.020348 | 0.151992 |
| gamma-Linolenic acid | 0.022875 | 0.151992 |
| Methionine | 0.023277 | 0.151992 |
| Phenylalanine | 0.023747 | 0.151992 |
| 1-Aminocyclopropane-1-carboxylic acid | 0.02701 | 0.151992 |
| Stearic acid | 0.028951 | 0.151992 |
| Itaconic acid | 0.029032 | 0.151992 |
| Glycine | 0.029355 | 0.151992 |
| Valine | 0.030368 | 0.151992 |
| Arachidonic acid | 0.031339 | 0.151992 |
| Glutamine | 0.04289 | 0.19811 |
| Leucine | 0.046952 | 0.207015 |

| Table S14 IUGR singletons vs sIUGR L (plasma) | | |
| --- | --- | --- |
| Metabolite | **p value** | **q value** |
| Palmitelaidic acid | 0.029553 | 0.394698 |
| Dodecanoic acid | 0.042911 | 0.394698 |
| Malic acid | 0.043894 | 0.394698 |
| Fumaric acid | 0.046526 | 0.394698 |
| bishomo-gamma-Linolenic acid | 0.048746 | 0.394698 |
| 4-Aminobutyric acid (GABA) | 0.049015 | 0.394698 |

| Table S15 IUGR singletons vs sIUGR S (plasma) | | |
| --- | --- | --- |
| Metabolite | **p value** | **q value** |
| bishomo-gamma-Linolenic acid | 0.033182 | 0.332914 |
| EPA | 0.039212 | 0.332914 |
| Dodecanoic acid | 0.043802 | 0.332914 |
| Palmitelaidic acid | 0.047186 | 0.332914 |
| Fumaric acid | 0.048668 | 0.332914 |
| Glycine | 0.049369 | 0.332914 |

| Table S16 Control vs sIUGR (placenta) | | |
| --- | --- | --- |
| Metabolite | **p value** | **q value** |
| Malonic acid | 0.003446 | 0.111168 |
| Pyroglutamic acid | 0.006235 | 0.111168 |
| Cyclopentasiloxane, decamethyl | 0.006827 | 0.111168 |
| N-alpha-Acetyllysine | 0.00783 | 0.111168 |
| Nicotinamide | 0.016332 | 0.182763 |
| Glutamine | 0.016776 | 0.182763 |
| Asparagine | 0.037135 | 0.315013 |
| Histidine | 0.037205 | 0.315013 |
| Nicotinic acid | 0.037591 | 0.315013 |

| Table S17 Control L vs sIUGR L (placenta) | | |
| --- | --- | --- |
| Metabolite | **p value** | **q value** |
| Nicotinic acid | 0.042192 | 0.847217 |
| Pyroglutamic acid | 0.043487 | 0.847217 |

| Table S18 Control L vs sIUGR S (placenta) | | |
| --- | --- | --- |
| Metabolite | **p value** | **q value** |
| Malonic acid | 0.002641 | 0.184962 |
| Nicotinamide | 0.0081 | 0.237931 |
| Succinic acid | 0.018511 | 0.378897 |
| 2-Aminoadipic acid | 0.022978 | 0.378897 |
| Cyclopentasiloxane, decamethyl | 0.037743 | 0.378897 |

| Table S19 Control S vs sIUGR L (placenta) | | |
| --- | --- | --- |
| Metabolite | **p value** | **q value** |
| N-alpha-Acetyllysine | 0.023384 | 0.998404 |
| Pyroglutamic acid | 0.029025 | 0.998404 |

| Table S20 Control S vs sIUGR S (placenta) | | |
| --- | --- | --- |
| Metabolite | **p value** | **q value** |
| Cyclopentasiloxane, decamethyl | 0.00492 | 0.474989 |
| Malonic acid | 0.008189 | 0.474989 |
| N-alpha-Acetyllysine | 0.01245 | 0.481395 |
| Nicotinamide | 0.024694 | 0.531884 |
| Adipic acid | 0.046846 | 0.620123 |

| Table S21 sIUGR L vs sIUGR S (placenta) | | |
| --- | --- | --- |
| Metabolite | **p value** | **q value** |
| Malonic acid | 0.025691 | 0.758891 |

| Table S22 IUGR singletons vs Control L (placenta) | | |
| --- | --- | --- |
| Metabolite | **p values** | **q values** |
| Creatinine | 0.010941 | 0.951868 |

| Table S23 IUGR singletons vs Control S (placenta) | | |
| --- | --- | --- |
| Metabolite | **p value** | **q value** |
| Creatinine | 0.034159 | 0.605233 |

| Table S24 IUGR singletons vs sIUGR L (placenta) | | |
| --- | --- | --- |
| Metabolite | **p value** | **q value** |
| Glycine | 0.028612 | 0.332096 |
| trans-Vaccenic acid | 0.035156 | 0.332096 |
| Behenic acid | 0.036042 | 0.332096 |
| Adrenic acid | 0.040147 | 0.332096 |

| Table S25 IUGR singletons vs sIUGR S (placenta) | | |
| --- | --- | --- |
| Metabolite | **p value** | **q value** |
| Adrenic acid | 0.021028 | 0.211157 |
| trans-Vaccenic acid | 0.021166 | 0.211157 |
| 2-Aminoadipic acid | 0.027094 | 0.211157 |
| Cyclopentasiloxane, decamethyl | 0.028683 | 0.211157 |
| 11,14-Eicosadienoic | 0.029245 | 0.211157 |
| 10,13-dimethyltetradecanoic | 0.030191 | 0.211157 |
| beta-Citryl-L-glutamic acid | 0.039055 | 0.211157 |
| Behenic acid | 0.039425 | 0.211157 |
| DHA | 0.042822 | 0.211157 |
| Margaric acid | 0.045272 | 0.211157 |
| Glycine | 0.049092 | 0.211157 |
